# A portable and high-sensitivity optical sensing system for detecting fluorescently labeled enterohaemorrhagic *Escherichia coli* Shiga toxin 2B-subunit

**DOI:** 10.1371/journal.pone.0236043

**Published:** 2020-07-16

**Authors:** Jeongtae Kim, Jun-Young Park, Young-Jun Park, Seo-Young Park, Moo-Seung Lee, Chiwan Koo

**Affiliations:** 1 Department of Electronics and Control Engineering, Hanbat National University, Daejeon, Republic of Korea; 2 Environmental Diseases Research Center, Korea Research Institute of Bioscience and Biotechnology, Daejeon, Republic of Korea; 3 Department of Biomolecular Science, KRIBB School of Bioscience, Korea University of Science and Technology (UST), Daejeon, Republic of Korea; Consiglio Nazionale delle Ricerche, ITALY

## Abstract

We developed a stand-alone, real-time optical detection device capable of reading fluorescence intensities from cell samples with high sensitivity and precision, for use as a portable fluorescent sensor for sensing fluorescently labeled enterohemorrhagic *Escherichia coli* (EHEC) Shiga toxins (Stxs). In general, the signal intensity from the fluorescently labeled Stxs was weak due to the small number of molecules bound to each cell. To address this technical challenge, we used a highly sensitive light detector (photomultiplier tube: PMT) to measure fluorescence, and designed a portable optical housing to align optical parts precisely; the housing itself was fabricated on a 3D printer. In addition, an electric circuit that amplified PMT output was designed and integrated into the system. The system shows the toxin concentration in the sample on a liquid crystal display (LCD), and a microcontroller circuit is used to read PMT output, process data, and display results. In contrast to other portable fluorescent detectors, the system works alone, without any peripheral computer or additional apparatus; its total size is about 17 × 13 × 9 cm^3^, and it weighs about 770 g. The detection limit was 0.01 ppm of Alexa Fluor 488 in PBS, which is ten thousand times lower than those of other smartphone-based systems and sufficiently sensitive for use with a portable optical detector. We used the portable real-time optical sensing system to detect Alexa Fluor 488–tagged Stx2B-subunits bound to monocytic THP-1 cells expressing the toxin receptor globotriaosylceramide (Gb3). The device did not detect a signal from Gb3-negative PD36 cells, indicating that it was capable of specifically detecting Stxs bound to cells expressing the toxin receptor. Following the development of a rapid and autonomous method for fluorescently tagging cells in food samples, the optical detection system described here could be used for direct detection of Shiga toxins in food in the field.

## 1. Introduction

Food-borne illnesses caused by various pathogenic bacteria, including *Escherichia coli*, *Salmonella*, and *Shigella*, are a growing public health concern worldwide. Although *E*. *coli* is a part of the normal intestinal flora of humans and animals, several pathogenic *E*. *coli* strains can cause human diseases. *E*. *coli* O157:H7 and other Shiga toxin–producing *E*. *coli* (STEC) are considered to be the most dangerous food-borne pathogens due to the severe symptoms associated with outbreaks [1]. Epidemiological analyses have associated specific STEC serotypes with both outbreaks and sporadic diseases ranging from hemorrhagic colitis to hemolytic–uremic syndrome (HUS). One of the most lethal STEC outbreaks occurred in Germany in June 2011: of 4000 cases, 845 of the patients suffered from HUS, and 53 died [2].

After ingestion of food or water contaminated with STEC, Stxs damage the intestinal tract leading to the systemic signs of Shiga toxemia and occasionally to extra-intestinal signs including microangiopathic hemolytic anemia, thrombocytopenia, and acute renal failure (which defines US), as well as acute encephalopathy. STEC strains carry two potent cytotoxins, Stx1 and Stx2, which are genetically and functionally conserved exotoxin proteins. Stx1 is genetically and structurally similar to the prototype toxin produced by *Shigella dysenteriae* serotype 1 strains. By contrast, Stx2 is only 56% similar to Stx1 at the amino acid level and is more cytotoxic when injected into mice or primates [3, 4]. In functional terms, Stxs have RNA N-glycosidase activity that inhibits eukaryotic protein synthesis. Structurally, the proteins adopt an AB5 configuration, consisting of monomeric enzymatic A subunit in non-covalent association with a pentameric ring of identical B subunits that bind to the toxin receptor Gb3 [5].

The Stxs produced by *Shigella dysenteriae* serotype 1 and select serotypes of *E*. *coli* are the most potent known virulence factors involved in the pathogenesis of hemorrhagic colitis, which can progress to potentially fatal systemic complications such as acute renal failure [6] and neurological abnormalities [7, 8]. Although multiple studies have defined the pathologic host responses to Stx1 and Stx2 in multiple cell types, sensors capable of detecting Stxs in susceptible cells following intoxication have also been recognized as critical tools (reviewed in [9, 10]). From the standpoint of public health, timely and accurate detection of STEC or Stxs in contaminated food, environmental samples, and patient tissue is key to preventing potential outbreaks. Several detection methods have been developed to date. The traditional culture method using Sorbitol–MacConkey agar (SMAC) is most commonly used for isolation of O157:H7 and O157:H- STEC, which are unable to ferment sorbitol [11]. Although SMAC is relatively inexpensive and effective, its sensitivity has become limited due to the emergence of non-O157 STEC strains that are capable of fermenting sorbitol. Conventional polymerase chain reaction (PCR) technology has been widely used to detect and identify Stx genes in direct extracts of foods and feces with extremely high sensitivity [11], but many first-generation PCR assays are labor-intensive, require time-consuming sample processing steps, and can only be performed by trained personnel; moreover, it is not clear whether these methods can distinguish between highly toxic EHEC strains and STECs with uncertain toxicity potential [12]. Enzyme-linked immunosorbent assays (ELISAs) for detection of Stx proteins are commercially available, but their sensitivities are generally lower than that of the Vero cytotoxicity assay; consequently, they are unable to detect low levels of Stx in samples [13]. Therefore, a rapid, simple, accurate, and sensitive identification technique is urgently required. To address this need, we explored the use of a toxin-detecting device with purified fluorescent Shiga toxin 2B-subunit as an *in vitro* model and then sought to miniaturize the device while maintaining high sensitivity.

Fluorescence detection has high sensitivity and precision, and has been applied in many fields. Several methods that can be used to detect fluorescence, including bulky microscopes, photomultiplier tubes (PMTs), avalanche photodiodes (APDs), charge-coupled devices (CCDs), and CMOS image sensors (CISs) [14–17]. Although these devices are sensitive, due to their large size they are not suitable for use in portable detectors. Various miniaturized and portable devices have been proposed, and several are in the development phase. Yao *et al*. described an ultra-small fluorescence detection system, sensitive at a wavelength of 518 nm, with dimensions of about 2 × 2 × 2 cm^3^, not including a signal reader or controller [18]. This system contains a super-bright LED as the light source and a photodiode with integrated preamplifier, and requires an additional device to acquire and process data. Pan *et al*. described a compact handheld LIF detector base on a quasi-confocal optical configuration [19]. To miniaturize the device, they used a small photodiode as an optical detector and the total dimension of their device was 9 × 7.5 × 7.7 cm^3^. Wang *et al*. presented a portable sensing platform using a smartphone [20]; the dimensions of the optical system were about 5 × 10 × 16 cm^3^, and the device was very low-cost (about $10). These devices were small enough to be portable, but had limited sensitivity and were unable to detect very weak fluorescence intensities, e.g., from low levels of Stx proteins on cells.

PMTs are more expensive than photodiodes or CMOS image sensors, but are much more sensitive than other optical sensors. This higher sensitivity allows a reduction in the volume of drugs or samples used in each experiment and simplification of the experimental steps; consequently, the overall cost does not differ significantly. Rather, as the number of experiments, analyses, and diagnostics increases in systems using photodiodes or CMOS image sensors, the volume of drug or substance tested must also increase. This can fully compensate for the greater expense of PMTs. In addition, a miniaturized device using an image processing–based sensor requires an additional high-performance processing device such as a computer or notebook for data processing. Optical detection systems using PMTs eliminate the need for additional computational equipment for signal processing, and due to their high sensitivity, they do not require amplification of low-concentration samples or use large quantities of samples. This decreases the time required for detection because no additional procedures are required, allowing quick inspection of the sample to be tested for.

In a previous report, we presented a real-time portable PCR system coupled with a highly compact fluorescence detector for the detection of fluorescently tagged amplified DNA [21]. Subsequently, we modified the optical detector component of that system and integrated it with a signal-amplifying circuit to increase its sensitivity. The entire system measures about 17 × 13 × 9 cm^3^ (width × depth × height) and weighs less than 770 g. In contrast to other fluorescence detectors that require a laptop/desktop computer and auxiliary instruments, the new device works with a battery-powered microcontroller unit, improving its portability and dramatically decreasing the overall cost of the system. As a proof of principle, we showed that the system was capable of detecting a fluorescent dye (Alexa Fluor 488) at concentrations between 0.01 and 0.1 ppm and dye-conjugated proteins at concentrations between 2 and 10 pg/μl of the Shiga toxin proteins.

## 2. Materials and methods

### 2.1. Optical sensing system

**Fig 1:**
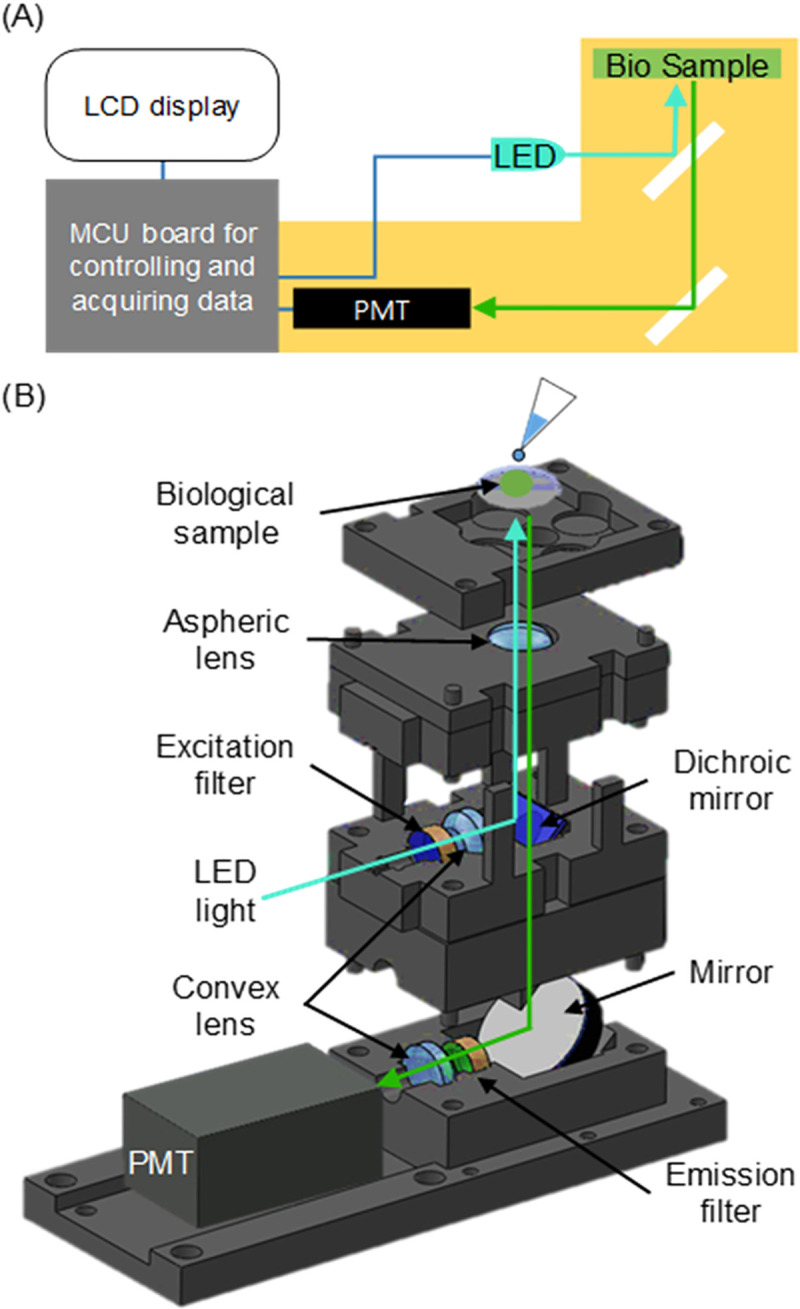
Schematic of the portable optical sensing system for fluorescently labeled bacterial Shiga toxins. (A) Configuration of the portable optical sensing system. The optical components (yellow box) perform fluorescence excitation and emission detection. The microcontroller unit (MCU; gray box) acquires the data and controls the electrical components of the system. The LCD shows the result of the measurement. (B) Construction of the optical sensing components.

The portable and real-time optical sensing system developed in this study consists of the optical components, the control and data acquisition circuits, and the display component (Fig 1A). The optical components detect fluorescence from a sample of Alexa Fluor 488–tagged Stx2B-subunits; the microcontroller unit (MCU) board, equipped with an amplifier, controls the system, acquires the data, and displays the result on an LCD.

The device contains three optical components: (1) the light source, (2) the platform for loading the Stx samples, and (3) the PMT for detecting the emitted light (Fig 1B). To excite Alexa Fluor 488 conjugated to protein, a cyan LED (LED490L, Thorlabs, Newton, NJ, USA) with a wavelength of 490 nm and FWHM of 20 nm was used as the light source. Light emitted from the LED is passed through an excitation filter (ET480/40x, Chroma Technologies, Bellows Falls, VT, USA) to eliminate unwanted light, and through a convex lens (LA1540A, Thorlabs) to focus filtered light. The focused light is reflected to the sample platform by a dichroic mirror (T510lpxrxt, Chroma Technologies) and excites the Stx sample on the sample platform. The aspheric lens (354330A, Thorlabs) is used to focus the emitted light from the fluorescent dyes. This emitted light goes through the dichroic mirror and is reflected to a compact photomultiplier tube (PMT) (H10721, 5 × 2 × 2 cm^3^, Hamamatsu, Iwata, Japan) by a mirror. The reflected light is passed through the emission filter (ET535/50m, Chroma Technologies) and a convex lens to eliminate unwanted light and focus the signal. The intensity of this light is detected by the PMT, within which the signal is amplified and converted to a current signal. The current output is converted to a voltage signal, and the voltage is acquired by the MCU (Arduino Mega 2560, Arduino, Ivrea, Italy), which shows the voltage data on a 3.5-inch TFT LCD display. The Arduino is an open-source platform that facilitates the use of multiple kinds of hardware and software. It provides a simple programming environment, and has been used in thousands of different projects, but is much less expensive than other microcontroller platforms. The voltage is correlated with the fluorescence intensity from the Stx sample.

#### 2.1.1 Optical housings

The optical detection system required precise setup of the optical components. To this end, the optical housing in which the optical components are placed must be fabricated with high precision. We fabricated our optical housings using a 3D material printer, which can print structures with a precision of tens of microns, at low cost, in a small amount of time. First, we designed the optical housings using 3D CAD tools.

As shown in Fig 1B, the bottom of the housings contains structures in which the PMT, emission filter, convex lens, and mirror can be placed. These structures were designed for aligning the center of the optical components so that the emitted light from the fluorescence sample entered the center of the PMT’s sensing area. In addition, the structure holding the mirror allowed the mirror to be placed at an angle of 45° in order reflect the emission fluorescence light from the sample into the PMT. The distance between the sensing region and the convex lens was designed based on the focal length of the convex lens. The distance between the convex lens and the mirror was the minimum possible given the length of the excitation optics.

The middle-layer housing was designed to hold the LED, excitation filter, convex lens, and dichroic mirror. These housings were designed so that the centers of all optical components were aligned with each other. The distance between the LED and convex lens was chosen based on the emission angle of the LED and the planar side area of the convex lens. The other distances were the minimum possible to accommodate the thickness of the walls holding the optical components. The structure where the dichroic mirror was placed was designed with the same angle as the mirror structure in the bottom housing. Moreover, the centers of the planar and dichroic mirrors were aligned.

Of the two top housings, one was designed to hold the aspheric mirror, and the other was designed to hold the glass chip for loading of the sample. The housing of the aspheric mirror was a rectangular structure with a circular hole larger than the diameter of the dichroic mirror. The sample cover housing was designed to block ambient light and facilitate sample loading. In addition, the outer housings were designed for blocking ambient light and fixing the housing layers in place. The designs and details of the housings are described in the S1 File.

#### 2.1.2 Electrical circuit

Although the PMT produces an amplified current signal, its amplitude can be as low as tens of microvolts. Because of the amplitude of the PMT’s signal, it needs to be amplified. Because a current signal is harder to measure than a voltage signal, it must be converted. To convert and amplify the current signal, we used a current-to-voltage converter with an operational amplifier (OP-AMP). The portable PMT used in our device has a reference output and control input pins that are used to control the gain of the portable PMT. For that purpose, a potentiometer was connected in series between the reference pin and the control input pin. The PMT output pin was connected to the OP-AMP, which was used to convert the current signal to the voltage signal and simultaneously amplify it. In a feedback line, a resistor and potentiometer were connected in series, and a capacitor was connected in a parallel with the resistor and potentiometer. The resistor, potentiometer, and capacitor were used to set the minimum gain, control the gain, and filter high-frequency signals of the amplifying circuit, respectively. Because higher-frequency signals have lower linearity, a low-pass filter was used to make the output linear. The output pin was connected to the MCU. More details about electrical circuits are provided in the S1 File.

#### 2.1.3. 3D printing

To print the housings, the designs of the housings were saved or converted to stereolithography (STL) file format that can be opened by the 3D printer software. In the 3D printer software, the STL files were converted to another format that is appropriate for the 3D printer, including printing settings. To make precision housings, the printing speed of the setting was set to 25 mm/s, and the printing layer height was set to 0.1 μm; lower speed and printing layer height result in higher precision. In addition, the inner filing value was set to 100% to remove the effect of ambient light.

### 2.2. Shiga toxins and sample preparation

#### 2.2.1. Toxins

Recombinant purified Stx2B-subunits were a kind gift from Vernon L. Tesh’s laboratory (Texas A&M University, College Station, TX, USA). The device developed in this study was used to detect purified Stx2B-subunit conjugated to Alexa Fluor 488 (ThermoFisher Scientific, Waltham, MA, USA). Thirty micrograms of purified Stx2B-subunits were labelled with Alexa Fluor 488 as described in the manufacturer’s protocol. THP-1 or PD36 cells were seeded at 1.0 × 10^5^ cells per well in four-well Lab-Tek chambered borosilicate cover glass slides (Nalge-Nunc International, Rochester, NY, USA), incubated overnight, and washed twice in complete RPMI-1640 growth media. In all experiments, to confirm the presence of Alexa Fluor–labeled Stx2B-subunits, images were acquired with an exposure time of 0.16 sec on an EVOS M5000 imaging system (ThermoFisher Scientific, Waltham, MA, USA). Within each experiment, all data were collected at identical settings.

#### 2.2.2. Samples preparation for evaluation

To evaluate the performance of our device, we conducted the fluorescence test with fluorescent dyes alone, as well as with dye-conjugated proteins. Dyes and proteins were used at concentrations of 0, 0.01, 0.02, 0.03, …, 0.08, 0.09, and 0.10 ppm and 0, 2, 4, 6, 8, and 10 pg/μl. To determine the sample concentrations, 1 μl of sample was dropped on a 2.54 × 2.54 mm^2^ glass slide, which was then loaded onto the platform of the optical housing. After the glass plate was loaded, the sample was excited with light from the 490-nm LED, and the emitted green fluorescent light was detected using the PMT. The detected light was converted to a voltage signal, and 10 voltage signal data were continuously acquired by the MCU. The collected voltage data were averaged and displayed on the LCD panel on the MCU board. In addition, to determine the availability of live cells for Shiga toxin detection, we performed tests on Gb3-expressing THP-1 and PD36 cells [22] (or parental cells not expressing Gb3, as negative controls) using poly-lysine. All measurements were repeated three times.

Cell culture was performed for the test of sensing amount of Shiga toxins in cells. The human myelogenous leukemia cell line THP-1 (American Type Culture Collection, Manassas, VA) was maintained in RPMI-1640 (Gibco-BRL, Grand Island, NY, USA) supplemented with penicillin (100 U/ml), streptomycin (100 mg/ml) and 10% fetal bovine serum (Hyclone Laboratories, Logan, UT, USA). Before stimulation, cells were incubated with RPMI-1640 containing only 10% fetal bovine serum. PD36 cells [22] were cultured in RPMI-1640 containing 10% fetal bovine serum, antibiotic–antimycotic, 0.1 mM MEM nonessential amino acids, 1 mM MEM sodium pyruvate, 55 μM 2-mercaptoethanol, and 2 mM L-glutamine. All cell culture was performed at 37°C and 5% CO2 in a humidified incubator. Complete growth media containing Alexa Fluor–labeled-Stx2 B-subunits were added to the cell monolayers on poly-L-lysine–coated glass slides. To clearly detect fluorescence, cells were washed extensively with sterile Dulbecco’s Phosphate Buffered Saline (DPBS, 1X, pH 7.4) to remove unbound proteins or possible sources of noise that could obfuscate results, and then imaged over the next 5–25 min.

## 3. Results & discussion

### 3.1. Fabrication

**Fig 2:**
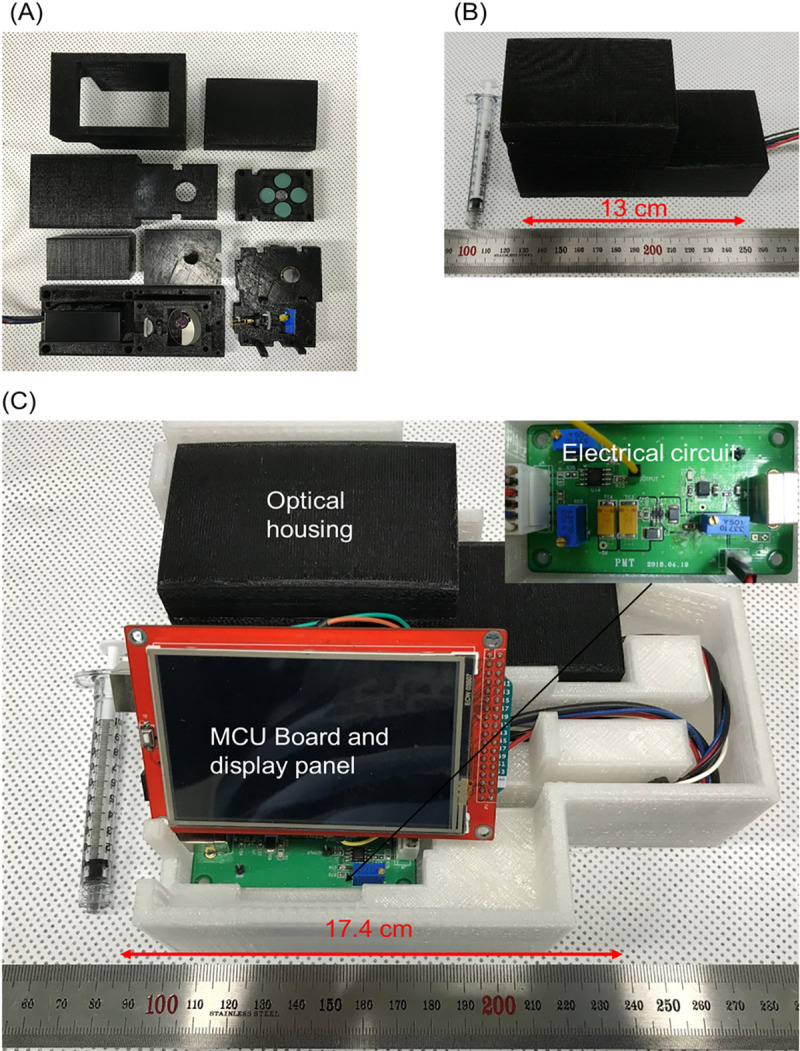
Fabricated portable fluorescence detector. (A) 3D-printed housing structures and optical components of the portable fluorescence optical detector. (B) Assembled optical housing structures. (C) Portable and real-time optical sensing system for detection of Stx2B subunits. The system consists of an optical housing, an MCU board with a 3.5-inch LCD display, and a circuit for amplifying electrical signals. The total size is 17 × 13 × 9 cm^3^ without the portable battery. The system functions without any auxiliary device.

The housing structures of the portable fluorescence optical detector were printed on an FDM 3D printer (3DISON Multi, 3DISON, Seoul, Korea); black PLA was used as the printing material (Fig 2A). Printing with the PLA filament was performed in the fully filled condition to prevent ambient light from passing through the printing material. Thus, stable output results were obtained during detection experiments in both dark and bright rooms. After the optical elements were placed inside the printed optical housing structures, the structures were assembled (Fig 2B). The electrical circuit on the PCB that processed the signal and the MCU that controlled the system and displayed the data were integrated with the optical housing and placed in a 3D-printed case (white), as shown in Fig 2C. The whole system was about 17 cm × 13 cm × 9 cm (width × depth × height) = 1989 cm^3^ in size and weighed less than 770 g. These dimensions are reasonable in comparison with several portable fluorescence detection systems, ranging in size from 7.6 cm × 7.6 cm × 12.7 cm (= 733 cm^3^) to 22 cm × 100 cm × 12.7 cm (= 11660 cm^3^) [23–27].

After our system was connected to a portable battery, it functioned without requiring any auxiliary device, and successfully detected fluorescence intensity from samples on the platform. Biological samples containing Stx were loaded on the platform after the top cover of the optical housing was opened.

### 3.2. Characterization using fluorescence

To evaluate the sensitivity of the detector system, we tested dilutions of Alexa Fluor 488 in PBS. Conventionally, the most common dilution factors for conjugation of Alexa Fluor 488 range from 1:300 to 1:1000, but we used higher dilutions ranging from 1:10^6^ (0.01 ppm) to 10:10^6^ (0.1 ppm). To detect lower concentrations of Stx proteins conjugated to fluorescent dyes, we optimized the detector system by using the maximum gain of the PMT device and increasing the gain of the current-to-voltage conversion circuit of the optical sensing device. We then characterized the system by testing very low concentrations of Alexa Fluor 488.

**Fig 3:**
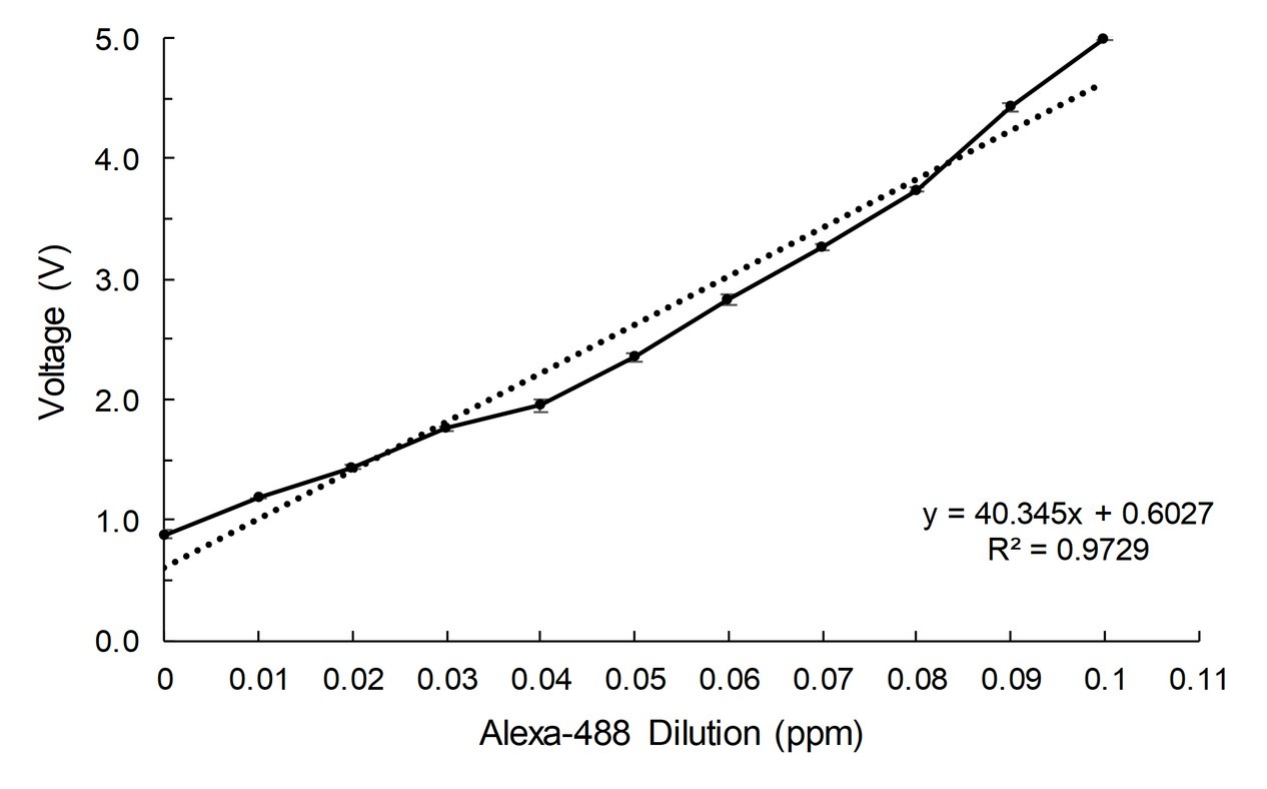
Correlation of fluorescent dye dilution and measured output voltage of the optical housing developed for this study. PBS alone (0 ppm) and the indicated dilutions of Alexa Fluor 488 were tested.

The measured voltage started at about 0.85 ± 0.05V in the absence of fluorescence (PBS-only sample) and increased linearly with the concentration of the fluorescent dye over the range from 0 to 0.1 ppm (Fig 3). Thus, the compact optical sensing device exhibited sufficient sensitivity at the lower limit of detection, as well as sufficient linearity to allow quantification. Our detection limit was 0.01 ppm, which according to the manufacturer’s manual, corresponds to 110 pM Alexa Fluor 488. Table 1 lists the detection limit and peripheral devices of several reported portable fluorescence detectors and our device together. Xin *et al*. developed a fluorescence detection device by fabricating a microfluidic system that used a pump together with an avalanche photodiode. That system had a detection limit of 150 pM, but requires a computer to analyze the measured signal [28]. Yao *et al*. developed a compact fluorescence detector using an optical sensor consisting of a shear amplifier and a photodiode. That device has a detection limit of 1.02 pM, but requires a precise multimeter for the measurement with a few milli volt orders, which may result in inaccuracies [18]. A device developed by Zhao *et al*., based on a cell phone and a CCD camera, has the advantage of being small but has a relatively low detection limit, 1.7 μM [29].

**Table 1:** Comparison of portable fluorescence detection devices.

	Proposed	Yao *et al*. [18]	Xin *et al*. [28]	Zhao *et al*. [29]
**Sensor Type**	Photomultiplier Tube	Photodiode and Preamplifier	Avalanche photodiode	CCD camera
**Peripheral devices**	None	High precision multimeter	Computer and pump	Smartphone
**Size (cm)**	17 × 13 × 9	2 × 2 × 2	Not reported (estimated: 40 × 80 × 70)	Not reported (estimated: 30 × 8 × 8)
**Weight (Kg)**	0.77	Not reported	Not reported	Not reported
**Power source**	Battery Powered	Not reported (estimated: AC + Battery powered)	Not reported (estimated: AC powered)	Not reported (estimated: battery powered)
**Detection limit**	110 pM	1.02 pM	150 pM	1.7 μM

### 3.3. Sensing the concentration of purified Stx proteins

**Fig 4:**
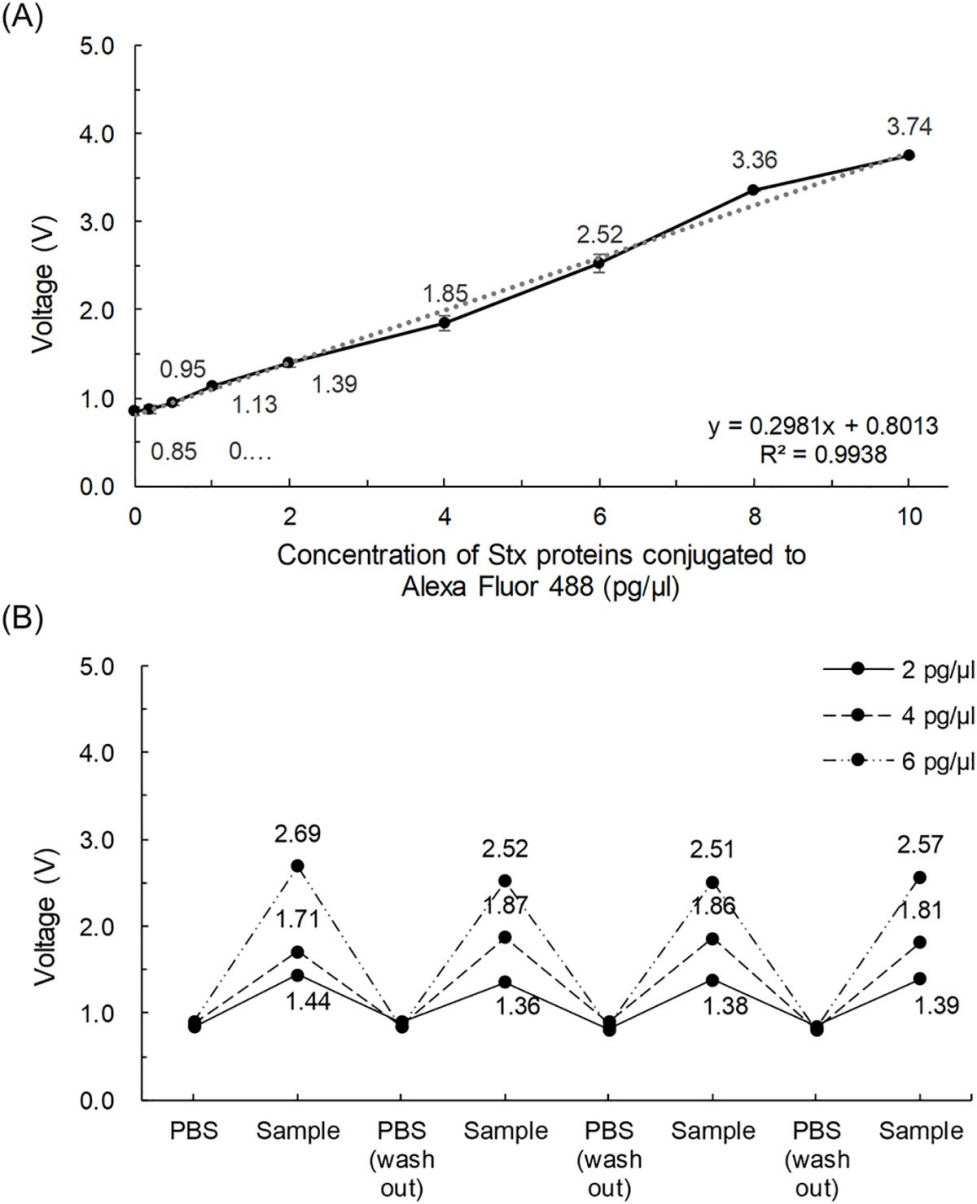
Performance test of a device for sensing labeled Shiga toxin proteins. (A) Correlation between concentrations of Stx2B conjugated to fluorescent dyes and output voltage of the portable fluorescence optical detector. (B) Repetition test. A sample (6 pg/μl) was measured, washed out with PBS, and measured again. The measurements were stable over four repetitions.

Fig 4A shows the results of measurements of different concentrations of purified Stx proteins conjugated to Alexa Fluor 488 (0, 2, 4, 6, 8, and 10 pg/μl Stx2B-subunit proteins). As the concentration of purified Stx protein increased, the fluorescent dye accumulated, and the emitted light intensity increased. Fluorescence detection exhibited good linearity in the range of 0 to 10 pg/μl (R^2^ = 0.97). Based on these results, our portable device was able to detect Stx with good linearity and high sensitivity in the picomolar range.

Fig 4B shows results of a repetition test. Ideally, the reading should be the same across repeated measurements, rather than depending on the number of repetitions. In this experiment, the fluorescent light intensities of Stx2B-subunits samples loaded on the glass substrate were measured repeatedly, and displayed in real time. Specifically, the light intensity from 2, 4, and 6 pg/μl samples was measured, and then the sample was washed out and measured again; this procedure was repeated four times. The relative standard deviation (RSD) of the measurement was less than 4.45%. In addition, the measurements were very similar across repetitions, indicating that our fluorescence optical detector yields stable measurements in real time.

### 3.4. Sensing test in THP-1 cells expressing the toxin receptor Gb3

**Fig 5:**
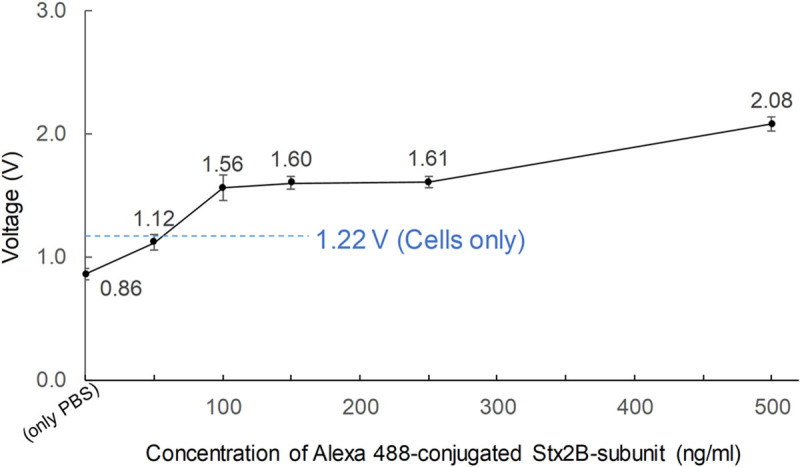
Performance of the detector using monocytic THP-1 cells expressing toxin receptor. Cells were cultured with the indicated concentrations of labeled Stx2B.

We then performed the detection test for Stx2B protein bound to THP-1 cells. The cells were cultured in the presence of 50, 100, 150, 250, and 500 ng/ml Stx2B-subunits conjugated to Alexa Fluor 488, and then rinsed with PBS buffer to remove the Stx2B-subunits that were not bound to cells. Each cell has a limited number of receptors where Stx proteins can attach, and not every Stx2B molecule bound to a cell. Therefore, the total number of Alexa Fluor 488 molecules was small, and collectively emitted a very weak fluorescent signal that would be difficult to detect using a normal portable optical sensor. Fig 5 shows the voltage output from our system using six different cell samples. The voltage signal (1.22 ± 0.05V) from the negative control (THP-1 cells not exposed to Alexa Fluor–labeled Stx2B-subunits) was considered as the threshold for detection of cells bound to Alexa Fluor 488–conjugated Stx2B-subunits. Voltages above this threshold value were obtained from samples of cells cultured with 100, 150, 250, and 500 ng/ml Alexa Fluor 488–conjugated Stx2B-subunits, indicating that the portable and real-time optical sensing system successfully detected the Stx2B-subunits bound to living cells. The 50 ng/ml sample was within the error of the threshold line, and was therefore considered below the detection limit of our system. The light intensity from the 100 ng/ml sample (1.56 ± 0.10 V) was similar to that of an Alexa Fluor 488 dilution of 0.035 ppm. Together, these results confirm that our system can detect low concentration of Shiga toxins with very high sensitivity.

**Fig 6:**
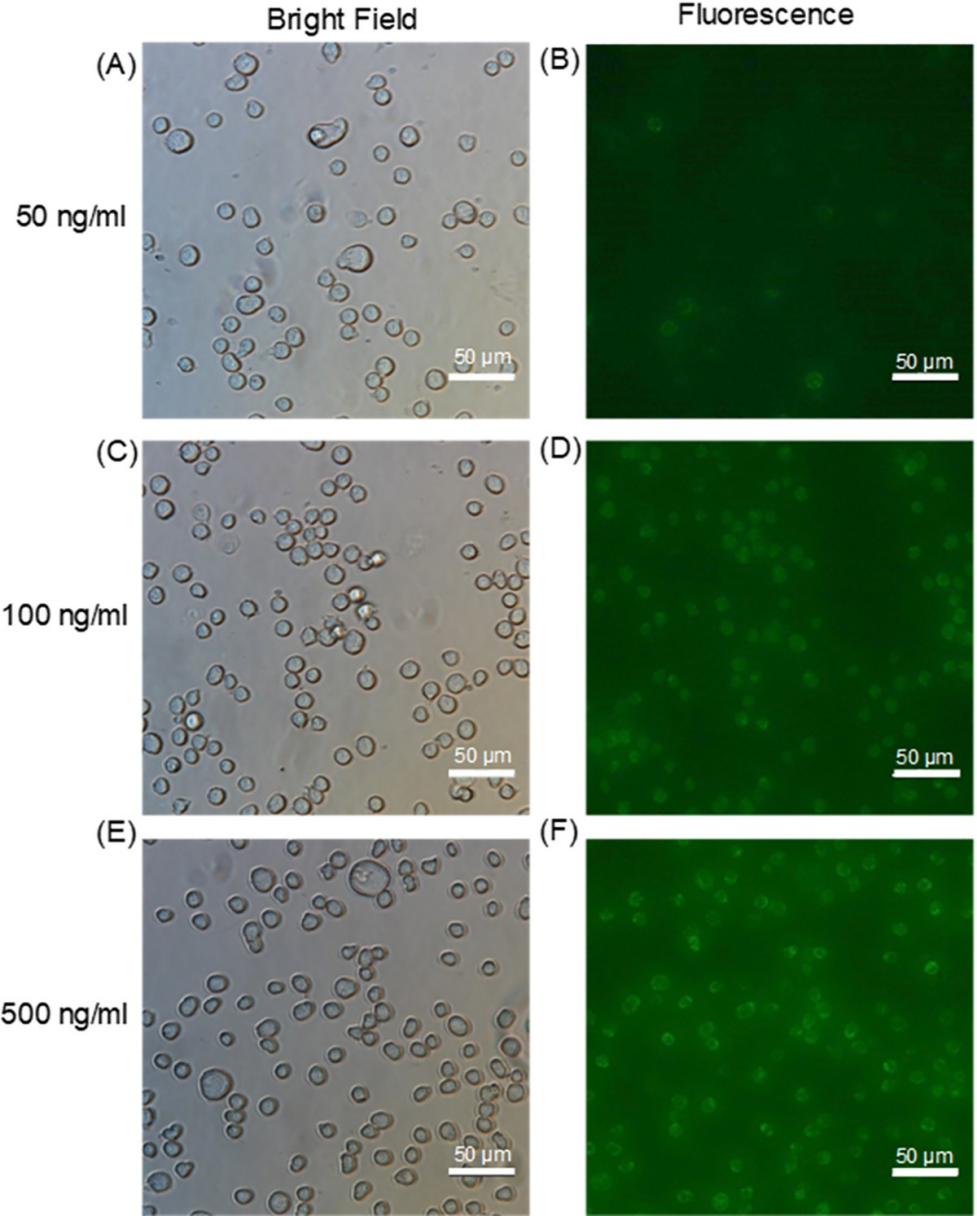
Fluorescence micrographs of fluorescently labeled Stx2B-treated Gb3-expressing THP-1 cells immobilized on the sensing device. Cells were cultured with 50 ng/ml (A–B), 100 ng/ml (C–D), or 500 ng/ml Alexa Fluor 488–conjugated Stx2B (E–F).

After the measurement test, we observed the cells under a fluorescence microscope to determine how many cells were bound to Stx2B-subunits (Fig 6). For cells cultured with 50 ng/ml Stx2B-subunits, the intensity of the fluorescence image was comparable to the background, as shown in Fig 6A and 6B. For cells cultured with 100 ng/ml Stx2B-subunits, the detection limit of our system, the intensity of the fluorescence image was brighter than the background (Fig 6C and 6D). Fig 6E and 6F shows bright field and fluorescence images, respectively, of cells cultured with 500 ng/ml Stx2B-subunits.

**Fig 7:**
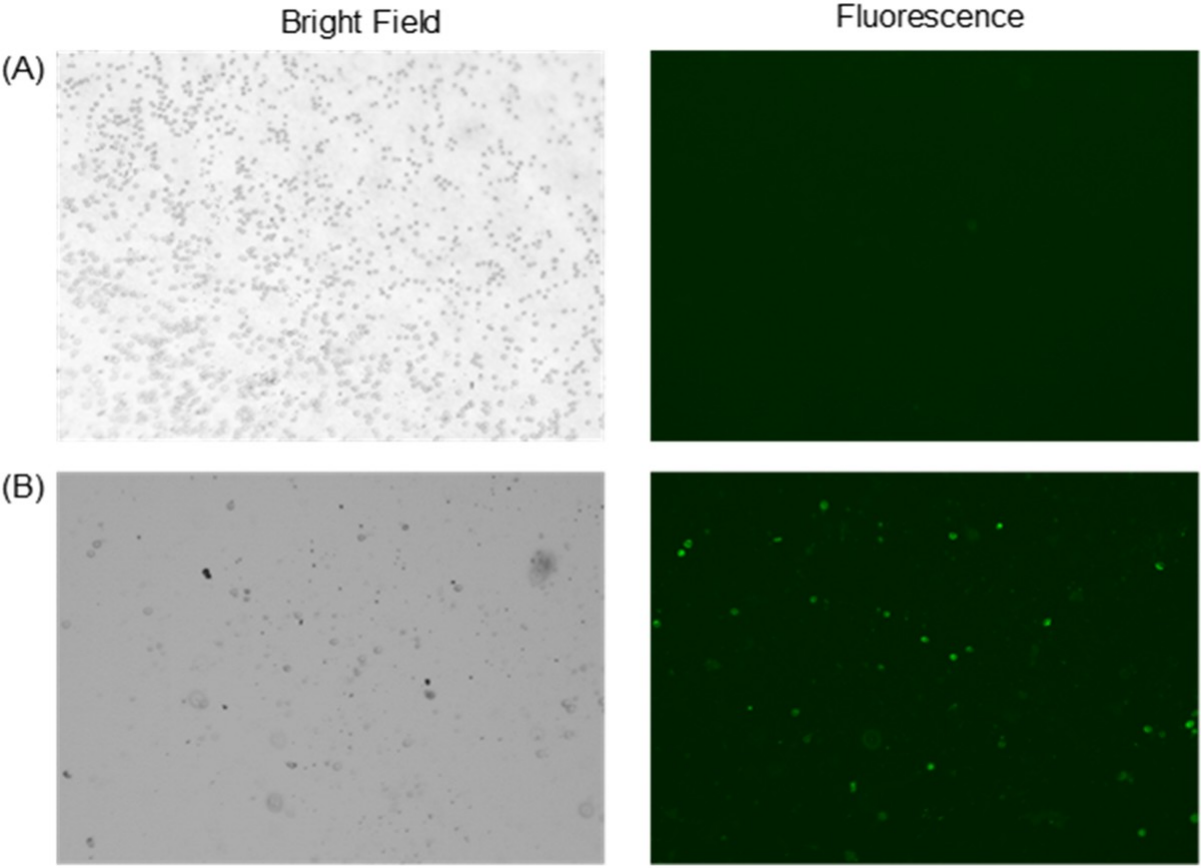
Fluorescence micrographs comparing THP-1 cells expressing Gb3 vs. PD36 cells not expressing Gb3. (A) PD36 cells did not bind Stx proteins and did not emit green fluorescence. (B) Monocytic THP-1 bound Stx proteins and emitted green fluorescence (bright green dots).

To confirm that the portable optical sensing system detects differences in Stx concentration rather than cell type, we used the device to measure monocytic THP-1 cells (which express Gb3 receptor) and PD36 cells (which do not) that had been cultured with Alexa Fluor 488–conjugated Stx2B-subunits. Using the portable optical sensing system, the voltage output from the PD36 sample was 0.90 ± 0.05V, indicating that no Stx2B-subunit was bound. With the monocytic THP-1 cells, the voltage was 1.41 V, above the threshold, indicating that the cells were bound to Stx2B-subunits. As observed by fluorescence microscopy, the toxin-sensitive monocytic THP-1 cells emitted green light whereas PD36 cells did not (Fig 7). The results of this experiment confirm that the system is sensitive to bacterial Shiga toxin protein concentration, but not cell type.

## 4. Conclusions

We developed a portable, real-time optical sensing system capable of detecting fluorescently labeled EHEC Shiga Toxin 2B-subunit, with the measurement expressed as a voltage output. The device consists of two main parts: the optical components and the data processing/display components. The system works alone, and does not require a peripheral computer or additional apparatus. The total size is about 17 × 13 × 9 cm^3^, and the weight is about 770 g, excluding a portable battery. To increase sensitivity, a PMT was selected as the optical sensor. The optical path was designed to allow excitation of Alexa Fluor 488 in biological samples and detection of emitted emission light from a fluorescence dye by the PMT. Accordingly, the optical housing was also designed to precisely align the optical components. The housing blocks were 3D-printed and assembled with an electrical circuit designed to amplify the PMT output signal and display the result on an LCD. The designs and details of the housings are described in the S1 File and all the parts are available online easily at a reasonable price. To evaluate the performance of the system, we conducted fluorescence detection tests using various dilutions of Alexa Fluor 488 in PBS. The system could detect fluorescence intensity from dilution as low as 0.01 ppm. Repetition testing confirmed that the measurements made by the system were reproducible. We then conjugated purified Stx proteins with fluorescent dye and tested samples containing various concentrations of Stx. Dye-conjugated Stx protein was detected with good linearity and high sensitivity, with a detection limit of 2 pg/μl Alexa Fluor 488–conjugated Stx2B-subunits. Finally, we tested the system’s ability to detect Stx on living cells that emitted weak fluorescence intensity. The device positively detected cells cultured with 100 ng/ml Stx2B-subunits. Collectively, these results show that our system is portable and capable of detecting small amounts of Shiga toxins. We anticipate that the system will have various potential applications in on-site food poisoning detection and other types of biological and medical analyses using fluorescence, and could also be adapted to a portable PCR system designed to detect small amounts of DNA.

## Supporting information

S1 File (DOCX)
