# Supplementary material for: A portable and high-sensitivity optical sensing system for detecting fluorescently labeled enterohaemorrhagic Escherichia coli Shiga toxin 2B-subunit
Source: PLoS One. 2020 Jul 16;15(7):e0236043. doi: 10.1371/journal.pone.0236043 (PMC7365435; doi:10.1371/journal.pone.0236043)
Supplement: S1 File — (DOCX) [file pone.0236043.s001.docx]

A portable and high-sensitivity optical sensing system for detecting fluorescently labeled enterohaemorrhagic *Escherichia coli* Shiga toxin 2B-subunit

**1. Optical housing**

The designs of the inner optical housings are shown in Supplementary Figure 1. Panel (A) shows the bottom housing. The optical components related to emission light can be placed in the bottom housing. The PMT was placed on the left side of the bottom housing, and the optical components on the right side. The distance of each optical component was set to the appropriate length with reference to the datasheets for the components. Red arrows indicate focal length, yellow lines indicate the center line of the optical components, and the green line indicates the sample position. In (A), the red arrow does not fit into the left wall of the structure that holds the components because the sensing region of the PMT is located below the surface of the sensing area. Panel (B) shows the excitation parts of the optical detection system. The distance between the LED and the convex lens was determined by the following equation.

$$L= \frac{D}{2tan \theta}$$

L is the distance between the emission point and the lens, D is the diameter of the planar area of the convex lens, and θ is the emission angle of the LED. The excitation filter was placed between the LED and convex lens. The structure in which the dichroic mirror is placed is located at the focal point of the convex lens, as the center will be aligned with the bottom mirror. The top parts consist of the aspheric lens and the sample substrate holder. The aspheric lens holder was just a hole with the same diameter as the aspheric lens.


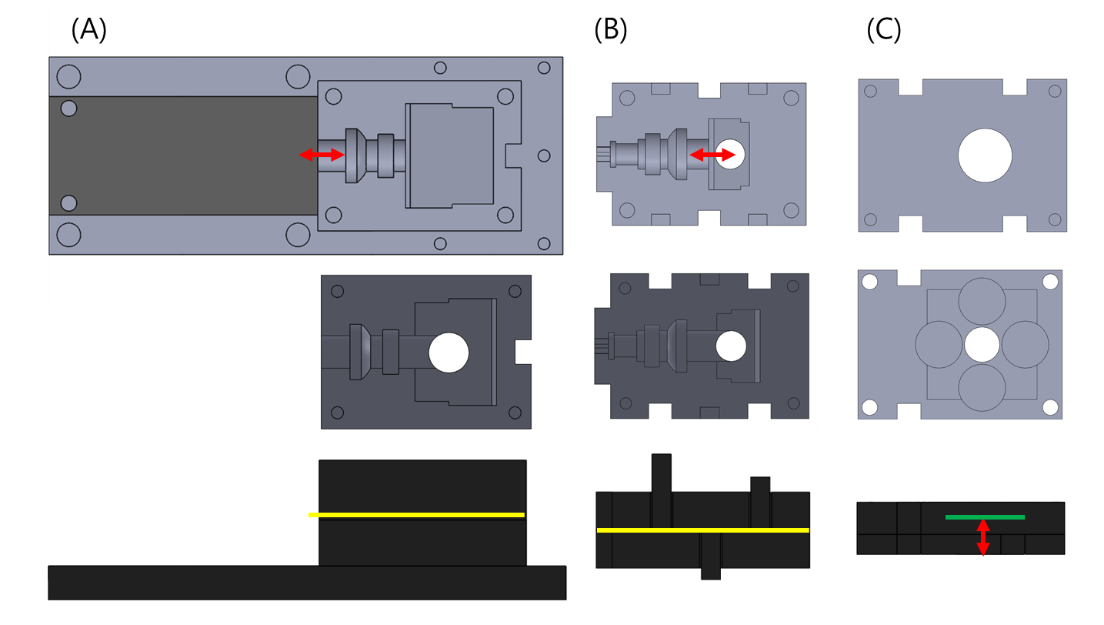


**Supplementary Figure 1. Designs of the inner housing of the optical detection system.** (A) Inner bottom housing for placing the PMT and the emission optics. (B) Inner housing for placing the excitation optics. (C) Inner housing for placing the aspheric lens and holding the sample substrate. Red arrows indicate the focal length of the lens and the green line indicates the position of the sample substrate.

The outer housings are shown in Supplementary Figure 2. If only the inner housing were used, ambient light might affect the measurement result because gaps exist the inner housing. Therefore, to remove the effect of light entering though the gaps, the outer housing was required. In addition, because the inner housings were easily disassembled, the outer housing was designed to hold the inner housing and block the effect of ambient light.


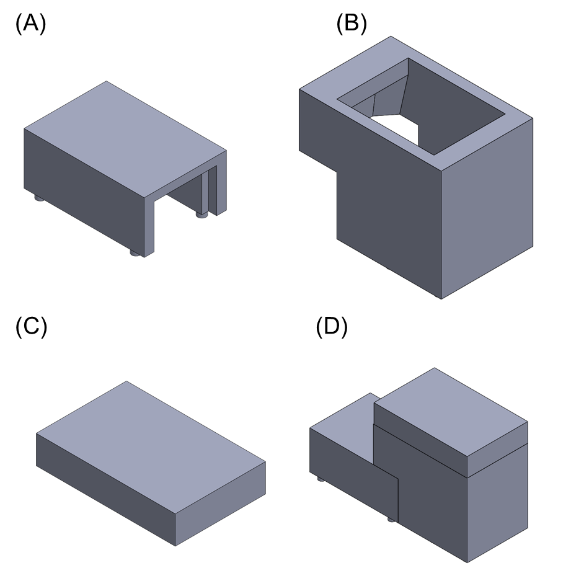


**Supplementary Figure 2. Designs of the outer housing of the optical detection system.** (A) PMT cover housing. (B) Outer housing for holding the inner optics housing. (C) Sample cover for protecting the sample from ambient light. (D) Assembled outer housing.

**2. Electrical circuit**

**2.1 Internal gain control circuit of the PMT**

The internal gain control circuit of the PMT is shown in Supplementary Figure 3. A potentiometer was connected to a voltage reference pin and a control input pin of the PMT in series. The voltage of the control input pin was determined according to the position of pin 2 of the potentiometer. The pin’s position can be set by turning a lever of the potentiometer. This internal gain control circuit operated as a voltage divider. The voltage of the control input was calculated by the following equation.

$$V_{CNT}= \frac{R_{23}}{R_{12}+ R_{23}} V_{ref}$$

$V_{CNT}$ and $V_{ref}$ are the voltages of the control input and reference output of the PMT, respectively. $R_{12}$ and $R_{23}$ are the resistance between pins 1 and 2 and pins 2 and 3 of the potentiometer, respectively. The control input of the PMT must be at least 0.5 V to operate, and according to the datasheet for the PMT, it should not exceed 1.1 V.


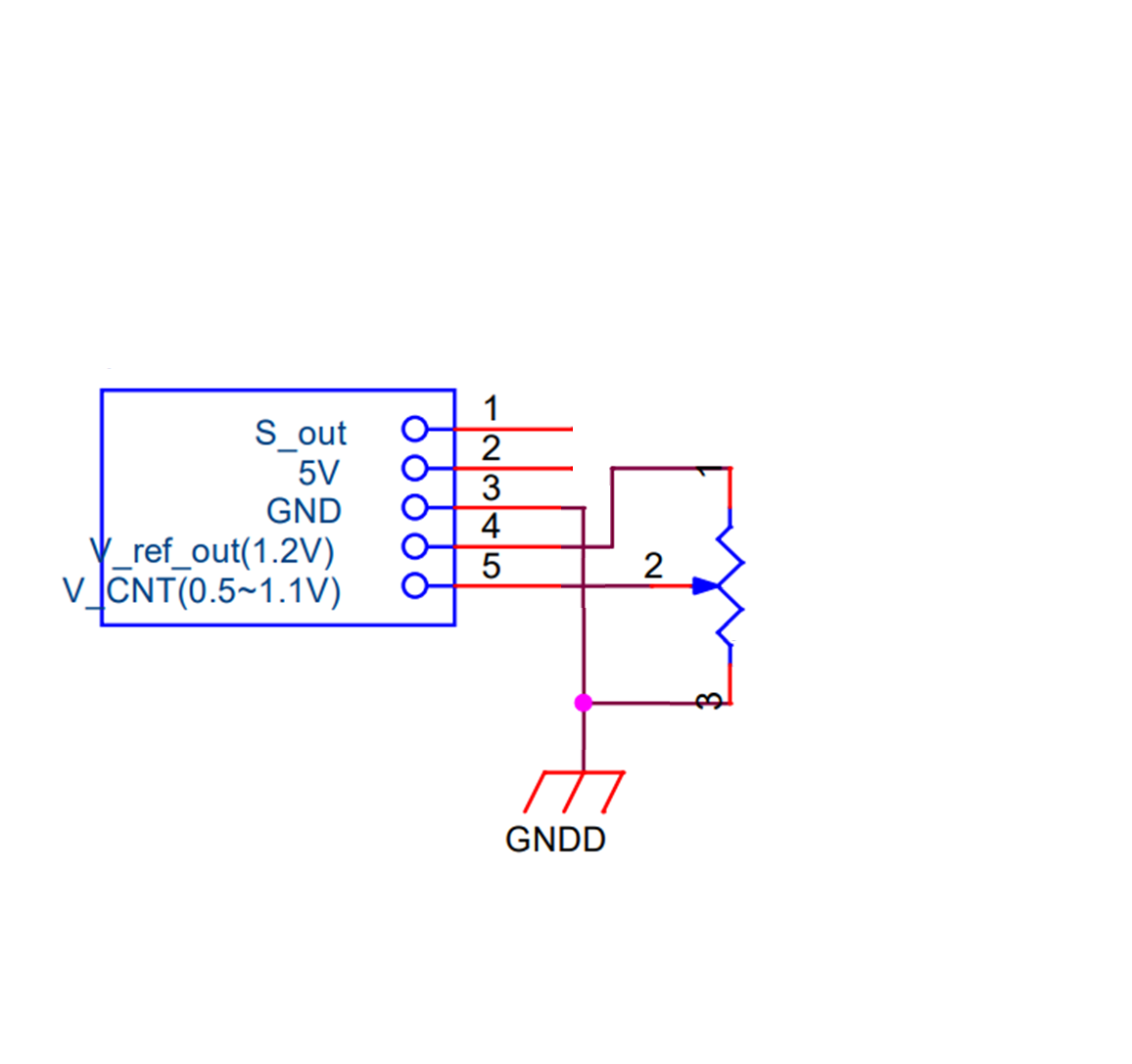


PMT

**Supplementary Figure 3. Internal gain control circuit of the PMT.** The potentiometer was connected to the 1.2-volt reference output pin and the control input pin in series. The control voltage was determined by the voltage between pins 2 and 3 of the potentiometer.

**2.2 Electrical circuit for reading and amplifying the PMT**

The electrical circuit for reading and amplifying the PMT is shown in Supplementary Figure 4; it is a current-to-voltage (I–V) converting circuit. In addition, the output signal of this circuit was amplified by feedback resistor R1. The I–V converting circuit produces an inverted signal, i.e., a positive-to-negative signal or negative-to-positive signal. Fortunately, because the output of the PMT was a negative signal, the inverted output from the converter was a positive signal. Consequently, another OP-AMP for inverting the signal was not required. The potentiometer to control the gain of the converting circuit was connected to the R1 resistor in series. In Supplementary Figure 4, the capacitor C1 is connected in a parallel with R1, and was used to filter high-frequency signals to produce linear output. The output was calculated by the following equation.

$$V_{out}= I_{PMT} R_{1}$$

$V_{out}$ and $I_{PMT}$ are the outputs of the converter and the PMT, respectively. The cut-off frequency of the converter was calculated as

$$f_{c}= \frac{1}{2\pi R_{1}C_{1}}$$

Although the cut-off frequency could be increased to acquire more signals, that would require another signal processing method involving a peripheral device. This could increase sensitivity at the cost of portability.

**
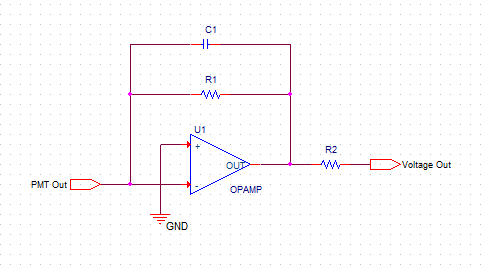
**

**Supplementary Figure 4. Circuit of the current-to-voltage inverting converter and amplifier.** Current is proportionally amplified with the feedback resistor. Output from the PMT is connected to the current input terminal. The voltage output terminal is connected to the input pin of the MCU board (Arduino Mega 2560). The output voltage read by the MCU is shown on the LCD display of the portable fluorescence optical detector.
